# Development of Coplanar Electro-Wetting Based Microfluidic Sorter to Select Micro-Particles in High Volume Throughput at Milliliter Amount within Twenty Minutes

**DOI:** 10.3390/s18092941

**Published:** 2018-09-04

**Authors:** Yuanyu Chen, Shauchun Wang

**Affiliations:** 1 Department of Chemistry and Biochemistry, National Chung Cheng University, Chia-Yi 621, Taiwan; curtis992250@gmail.com; 2 Center for Nano Bio-detections, Advanced Institute of Manufacturing with High-tech Innovations (AIM-HI), National Chung Cheng University, Chia-Yi 621, Taiwan; 3 Center for Innovative Research on Aging Society (CIRAS), National Chung Cheng University, Chia-Yi 621, Taiwan

**Keywords:** electrowetting-on-dielectrics, microfluidics, food safety pathogen, fluorescence detection

## Abstract

This paper reports the work of developing one coplanar microfluidic sorter while using the electro-wetting on dielectrics (EWOD) technique. When connected with delivery capillary to receive sample solution containing micro-particles, this device can select about 10 micro-particles in high volume throughput of milliliter amount within 20 min, to potentially match the requirement of efficiently determining the low amounts of bacteria in concentrated food and environmental samples, of which the typical bacteria density is 10 colony forming unit or less, much smaller than that of clinical pathogen samples. This coplanar T-shape EWOD device contains two fluidic channels, one inlet channel and the other collection channel stemmed from the middle of inlet channel. When the solution droplet falls from the delivery capillary to the entrance end of inlet channel, the droplet is driven to the intersection of two channels. The droplet containing fluorescent particle will be diverted to the lower channel to collect. Otherwise, the non-fluorescent droplet keeps moving toward the other end of inlet channel to waste zone. The particle fluorescence is collected through microscope lens to detect with one photomultiplier tube. The detected signals trigger the personal computer control board to active each EWOD electrode to direct the droplet moving directions. When the solution of 1 mL containing about 10 fluorescent micro-particles is delivered into this sorting device, nearly all the particles were correctly directed into collection zone in 20 min.

## 1. Introduction

Unlike conventional miniaturized lab-on-a-chip (LOC) devices performing assays in continuous microchannel fluidics, the alternative systems, such as electrowetting-on-dielectrics (EWOD) platform, which move discrete droplets on a substrate, have recently attracted increasing attentions because of the benefits to control fluid without using pumps and valves [1]. Besides, clogging, which frequently happened in microchannels, is not an issue. Therefore, EWOD devices have been successfully utilized in various bioanalysis applications, including clinical diagnostics and point-of-care tests [2,3], cell culturing [4], and biosensing [5].

Electrowetting is a phenomenon of altering the liquid wettability on a solid surface by applying external voltage to produce an electric field across the liquid-solid interface [6]. Such a wettability change is achieved by electric field-induced charge accumulation at the interface, causing the decrease of the associated interfacial tension γ_SL_.

To balance the forces between γ_SL_, γ_LG_, and γ_SG_, representing the interfacial tension between solid-liquid, liquid-gas, and solid-gas interfaces, respectively, existing at the edge of a sessile droplet, the contact angle θ therefore becomes external voltage (V) dependent, as described by electrowetting equation, as follows,

cos[θ(V)] = cos[θ(0)] + (C_H_/2γ_LG_)V^2^ (1)

where C_H_ is Helmholtz capacitance per unit area of solid-liquid interface.

EWOD is a popular design to implement electro-wetting device in which an insulating layer separates the working liquid and actuation electrodes [6]. Although considerable voltage (~100 volts) is needed to apply on the electrodes, EWOD has a practical advantage using insulators to guard working fluids from electrodes, thereby allowing for a much higher electric field to result in stronger electro-wetting effect before an electrical leakage or breakdown occurs.

Using the EWOD device droplet actuation can be achieved by switching the voltage “on” and “off” between a series of adjacent electrodes in parallel, resulting in an interfacial tension gradient. The tension difference across the actuated droplet can be used to individually manipulate droplets on a solid substrate without using moving parts.

EWOD devices are realized as two types of chip configurations. The first type is the coplanar configuration device, of which the ground electrodes are positioned in the same (bottom) plane as that the actuation electrodes are printed on. The sessile droplets moving on the coplanar EWOD chip directly contact with air. The other type of closed chip configuration uses two planes to sandwich manipulated droplets between the actuated electrode plane and the ground electrode plane.

EWOD devices typically assay samples at the volume between pL to μL in a couple of hours [7,8]. This volume throughput is adeqaute at clinical and biopharmaceutical research settings, where in particular reagent consumption is a cost concern. However, to implement EWOD methods to determine real world samples, especially of environmental monitoring and food safety applications, the aforementioned volume through at µL cannot handle these real samples at the volume level of mL or larger.

To overcome this technical chanllenge to interface the large volume sample that was delivered from continuous flow conduit into EWOD chip handling discret drops, that is “analog-to-digital interfacing” problem, two types of designs have been reported. The first design is using perconcentration unit to carry out magnetic bead-based or immunoprecipitation enrichment protocols [7,8,9,10]. The other design is to fabricate programmable parallel channels to divert sample solutions into several conduits prior to dispensing drops into the EWOD chip [11]. These designs apparently incease the system complexity of EWOD platforms.

Because of the open configuration, the coplanar EWOD chip alleviates the need for a cover plate. As a result, the coplanar EWOD chip can provide higher volume throughput than the two-planar chip. Senez’s group have developoed one analog-to-digital fluidic converter able to dispense 2 mL DI water into ~1.5 µL droplets passing through co-planner EWOD chip within 30 min, proving the potential of using co-planner chip to assay real world samples [12]. In addition to the device simplicity, the coplanar chip is indeed a suitable choice to be used in determining environmental and food samples, in which the sample volumes are usually at the level of milliliters. Currently the strict regulatory standards of water and food safety only allow for extremely low or even non-detectable levels of pathogen bacteria, such as *Salmonella* and *Campylobacter,* in tested samples in many countries [13]. Therefore, the quantitation limits of testing methods have to be down to several or even single copy of bacteria to assure the food safety. Besides, under the condition to detect such a limited numbers of bacteria copies, the sizes of samples in volume or weight also have to be large enough to obtain valid results. Currently, tedious cultivation procedures are still needed to enrich bacteria density prior to the determinations typically using the polymerase chain reaction-based methods.

Therefore there remains a need to develop highly sensitive techniques to determine bacteria counts in a timely manner. This technique should be also capable of assaying considerable volumes of each sample, at least 1 mL equivalent to the volume of typical concentrated sample for food safety analysis. While using fluorescent particles to mimic fluorophore-labeled bacteria, we develop a coplanar EWOD device to efficiently sort out and collect ~10 particles in sample solution of 1 mL.

## 2. Materials and Methods

**Figure 1:**
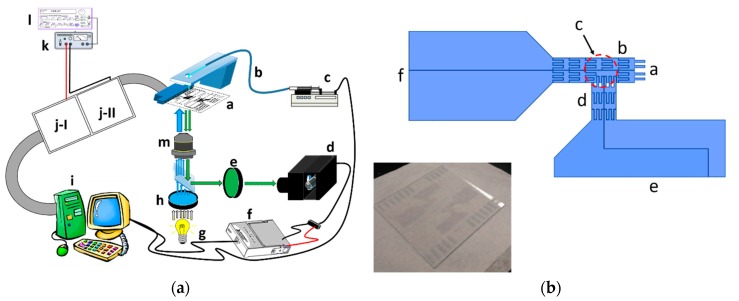
(**a**) the schematic illustration of coplanar electro-wetting based microfluidic sorter. a. electro-wetting-on-dielectrics (EWOD) chip; b. sample delivery capillary; c. syringe pump; d. photomultiplier tube; e. bandpass filter (532 nm); f. digital input/ output interface card (NI 9215 DAQ); g. mercury lamp; h. bandpass filter (488 nm); i. personal computer with digital input/ output interface card (NI 6509 DAQ); j. relay array units I and II; k. high voltage amplifier; l. function generator; m. microscope objective (10X); and, (**b**) the schematic illustration of EWOD chip layout. a. sample droplet entrance; b. inlet channel; c. detection zone; d. collection channel; e. droplet collection zone; f. droplet waster zone. The imbedded image at the lower left corner is one photograph showing two sets of this EWOD device printed on one slide.

Figure 1a illustrates the experimental set-up, in which the EWOD chip was stationed on the inverted fluorescence microscope stage to collect the fluorescent micro-particles that were contained in the sample solution, dripped into the chip as individual droplets.

These pins received controlling signals from the breadboard, where the control circuits were built, using connector wires. Using a functional generator and a voltage amplifier, the actuation voltage signals were sent to the control circuit. The relays in the breadboard were activated or disabled by the input/output interfacing card in the personal computer to control the voltage applications on each electrode.

The fluorescent micro-particle suspension solution was pumped through a glass capillary while using a syringe pump, controlled by the personal computer to drip the droplets on the inlet of EWOD channel.

The fluorescence excitation was using blue light at 488 nm with mercury lamp to illuminate the micro-particles though a filter and a microscope lens. The emission fluorescence collected from the same lens was detected with a photomultiplier (PMT) through a band pass filter of 532 nm. The voltage signal of PMT was obtained with the data acquisition card (DAQ) into personal computer as the triggering signals of control circuit.

The graph in Figure 1b shows the design of our EWOD device, consisted of two channels in T-shaped layout. The sample solution was continuously driven using a syringe pump through one delivery capillary. The droplets were dripped from the capillary end onto the entrance end of the EWOD chip inlet channel (circled with red dash line). The electrode sets of inlet and collection channels were connected with two relay units separately as two independent circuits. In the default settings, the EWOD electrodes of inlet channel were activated by the computer controlling card through one relay array unit to drive one sessile droplet dripped on the entrance toward the cross section of two channels. This droplet then stayed at the detection zone above the objective lens of the inverted fluorescence microscope (marked with one dashed rectangle in dark red). The collection channel electrodes remained idle at the same time. When one droplet containing fluorescence micro-particles was moved to the detection zone, the fluorescence emission was collected through the objective to strike on PMT. The PMT output signal was sent to the computer interfacing card to initiate the droplet direction diversion action. This fluorescent droplet was driven into the collection channel. In practice, when the intensity of this PMT signal was greater than a pre-set threshold level, the other relay array unit was triggered by the interfacing card to activate the collection channel electrodes. The previously activated relay array unit of inlet channel was shut down to disconnect the control electrodes at the same time. Because the inlet channel electrodes were no longer functioning, the other electrode set was able to pull the droplet in the detection zone into the collection channel. When there was no fluorescence emission from the droplet in the detection zone, the inlet channel electrodes remained activated to keep driving the droplet into the waste zone, while the collection channel electrodes continued to remain idle (the photograph of two sets of this EWOD device printed on one slide is shown in the imbedded image in the lower left corner of Figure 1b). The photolithography-based fabrication procedures of EWOD device are described in Appendix section.

**Figure 2:**
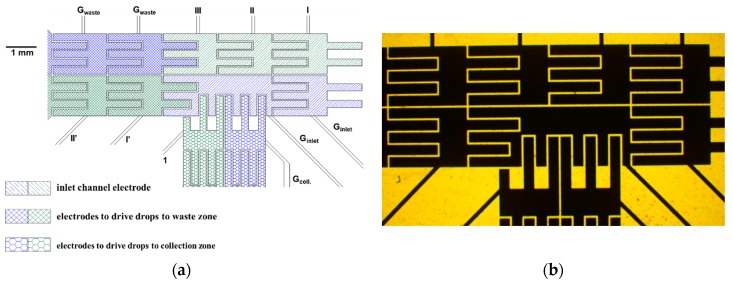
(**a**) The diagram showing the electrodes performing EWOD sorters. Electrodes I, II, and III (filled with green oblique line patters): activation electrodes to drive droplet from the inlet to the detection zone. Electrodes I’ and II’ (filled with green mesh patterns): activation electrodes to drive droplet from the detection zone to the waste zone. Electrode 1 (filled with green honey cone patterns): activation electrode to divert the droplet into the collection zone. G_inlet_, G_waste_, and G_coll_. Filled with blue patters: ground electrodes of the aforementioned three channels; and, (**b**) the image of photolithography mask to fabricate EWOD sorter electrode.

The Figure 2a is the diagram showing the activation and ground electrodes near the detection zone at our EWOD chips. The delivered droplet falling on the first set of electrodes from the right hand side of inlet channel (filled with oblique line patterns) was driven into the detection zone by activating electrodes I, II, and III in this diagram. These three electrodes and the other two adjacent ground electrodes were shut down. When PMT collects fluorescence emission from the droplet in the detection zone, the activation electrode 1 of collection channel (filled with honey cone patters) was on to divert the droplet toward collection zone. Otherwise, the electrodes I’ and II’ (filled with mesh patters) were activated to keep driving the droplet into the waste zone. The activation voltage profile applied on the electrodes to perform the aforementioned tracks were sine waveform of 200 Vrms of 1 kHz. The image of photolithography mask to fabricate these electrodes near the detection zone is provided in the imbedded graphy in Figure 2b.

The video clips, “direct advancement” and “diversion”, are provided in Supplementary Materials. The clip “direct advancement” shows the droplet movement trajectory on the EWOD chip along the inlet channel when the control electrodes were in default settings. The clip “diversion” shows that when the electrode set of collection channel was triggered, the direction diversion of droplet at the cross section of two channels was initiate. This drop was then driven toward the vertical direction into the other channel.

## 3. Results and Discussion

**Figure 3:**
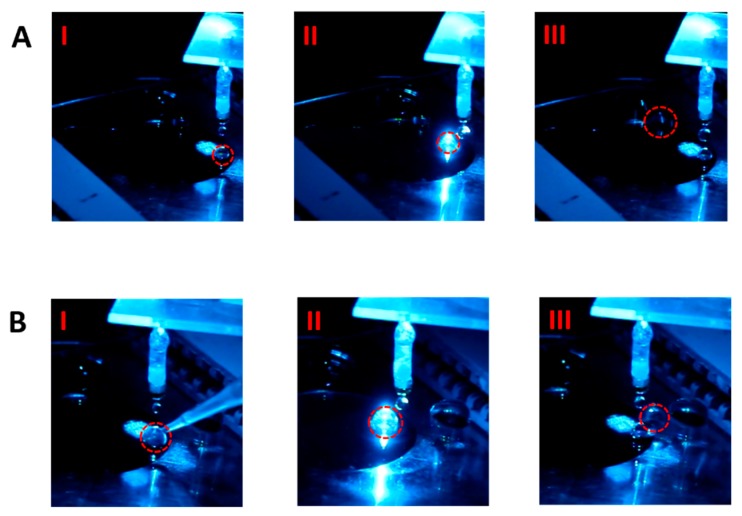
Droplet advancement images. (**A**) Non-fluorescent droplet. Snapshot I, droplet dripped at the inlet channel entrance; snapshot II, driven to the detection zone; snapshot III, reached the waste zone; and, (**B**) fluorescent droplet. snapshot I, droplet dripped at the inlet channel entrance and filled with fluorescent microparticles via a pipette tip; snapshot II, driven to the detection zone to emit fluorescence; snapshot III, diverted to the collection channel.

The photos in Figure 3A show the advancement of one non-fluorescent droplet toward the waste zone along the inlet channel of this EWOD device. The snapshot I was obtained when the droplet was dripped at the inlet channel entrance. In the snapshot II when the droplet was driven to the detection zone to glow, because no fluorescence signal was detected, the droplet continued to move forward. Finally, the droplet reached the waste zone (snapshot III).

One the other hand, in Figure 3B, because the fluorescence particles were added into the droplet dripped at the channel entrance (the snapshot I), when the droplet was driven into the detection zone, the intense fluorescence signal clearly appeared upon the incident light illumination through the microscope lens (snapshot II). The EWOD electrodes were therefore actuated to divert the droplet vertically into the other channel when the fluorescence signal was received to trigger the electrode control circuit. The diverted droplet was finally dragged to the collection zone (snapshot III).

**Figure 4:**
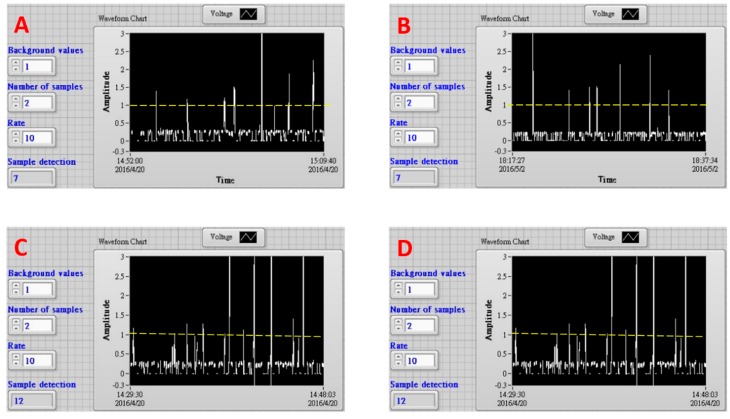
Temporal fluorescence signal traces during droplet sorting processes using EWOD device. Frame (**A**): sample aliquot containing about 10 fluorescent microspheres (1000 μL); Frame (**B**): the same sample solution as that in A of 1200 μL aliquot; Frame (**C**): sample aliquot containing about 25 fluorescent microspheres (1200 μL); Frame (**D**). the same sample solution as that in C of 400 μL aliquot. The dashed lines in each frame are the cut-off level to actuate the EWOD electrode sets to divert the droplets.

One aliquot of fluorescent microsphere solution (1000 μL to 1200 μL) containing about 10 microparticles was delivered into EWOD device with a syringe pump through a needle. The sample aliquots were dripped into the device as 100 droplets approximately, which were driven in a one-by-one manner to the detection zone to sort prior moving into waste or collection zone. The traces in Figure 3A,B show typical fluorescence signals that were acquired from the detection zone in the EWOD device. The dashed lines in these frames were the cut-off level to actuate the EWOD electrode sets to divert the droplets. This cut-off level was decided using the highest flicker noise spike peak intensity in one scattering signal trace using one blank solution containing no fluorescence micro-particles (data not shown). There were eight signal spike peaks shown in Figure 4A. Only one of these eight peaks did not reach the cut-off intensity, which is one highly possible flicker noise spike. The variations of peak intensities were attributed to microsphere aggregation as dimers. The electrode sets were triggered seven times to divert fluorescent droplets. Finally, 12 fluorescent microspheres were found in the collection zone. One fluorescent microsphere was found in the waste zone. Similarly, there were seven peaks found in the trace of Figure 4B. The electrode sets were triggered seven times. Eight fluorescent microspheres were found in the collection zone, while no particle was found in the waste zone. These results demonstrate that the fluorescent microspheres were sorted by our EWOD device with high accuracy. Nearly all of the fluorescent particles were accurately diverted from the inlet channel into the collection zone.

The other aliquot of 1200 μL containing about 25 fluorescent microspheres was also delivered into the device to collect micro-particles. The fluorescence signal trace was shown in Figure 4C. The electrode sets were turned on 12 times. There were 21 fluorescent microspheres found in the collection zone. In this condition of higher particle density, most microspheres were found to aggregate as dimers in droplets. On the other hand, four microspheres were found in the waste zone. Three or two fluorescent micro-particles of individual microspheres or dimer aggregates were assumed mistakenly move into the waste zone because their fluorescence signals were not detected. Although this efficiency of collecting 12 but missing three micro-particles out of 15 ones in total was acceptable, the sorting accuracy started deteriorated. The other sample of similar particle density and volume was also dripped into this EWOD device. The less efficient collection results were obtained too. The electrode sets were actuated 10 times. There were 15 fluorescence micro-particles were found in the collection zone and another three micro-particles were in the waste zone.

Figure 4D shows the fluorescence signal trace using one smaller aliquot (400 μL) of similar particle density to that of sample in Figure 4C. This sample was dripped into the device as 38 droplets. The electrode sets were triggered six times to divert fluorescent droplets. Eight fluorescent microspheres were finally found in the collection zone, while no particle was in the waste zone. The high sorting accuracy was resumed when one smaller sample aliquot was used. The aforementioned microsphere collection results are also listed in Table 1.

## 4. Conclusions

We successfully develop one EWOD device to count the numbers of fluorescence microparticles that are contained in one sample of 1 mL. When there are less than 10 fluorescence microparticles, which are either individual microspheres or dimer aggregates, contained in the sample, our EWOD device can collect the micro-particles with high accuracy. One the other hand, when the numbers of particles or aggregates are greater than 10, the sorting accuracy decreases, although the collection efficiency is still acceptable. This device has potential to determine the numbers of food safety pathogen bacteria without tedious culturing procedures.

To handle real samples containing bacteria, of which the typical sizes are only a few microns smaller than that of the microspheres used in this study, a couple of possible strategies are being developed in our laboratory as follows. First, functionalized magnetic nanoparticles bound on the detected microspheres or bacteria are employed to accumulate them close to the top of each droplet with magnet. In addition, the development image recognition algorithm is in progress to replace PMT detection to avoid false negative situations [14].

## Figures and Tables

**Table 1:** Particle detection results using the co-planner EWOD device developed in this work.

	Sample Volume (µL)	Triggering Occurrence	Numbers of Fluorescent Microspheres in Collection Zone	Numbers of Fluorescent Microspheres in Waste Zone	Estimated Numbers of Detected Particles	Estimated Numbers of Missed Particles	Estimated Total Numbers of Particles
#1	1000	8 times	12	1	8	1	9
#2	1000	7 times	8	0	7	0	7
#3	1200	12 times	21	4	12	3	15
#4	1000	10 times	15	3	10	2	12
#5	400	6 times	8	0	6	0	6

## Supplementary Materials

The following are available online at http://www.mdpi.com/1424-8220/18/9/2941/s1, Video S1: droplet movement trajectory.

## Appendix A. EWOD Chip Electrode Fabrication Procedures

Step 1. Glass chip cleaning: Indium Titanic Oxide (ITO) glass chip was rinsed with acetone, methanol, and then DI water to wipe out greasy stains on the surface. Next nitrogen gas was used to dry down liquid deposits. Finally the glass chip was placed on the heating plate to completely remove liquid residues to ensure the surface cleanness.
Step 2. Photoresist coating: Having been cooled down, the dried chip was coated with positive photoresist materials such as S1813 using a spin coater. The inspections of surface cleanness and flatness were performed to avoid coating defect and dust contaminations. Finally the coated chip was placed on the heating plate to bake at 90 °C for 5 min.
Step 3. UV exposure: When the coated chip was cooled down, the patterns of plastic mask were transferred to the positive photoresist coating layer using UV exposure method. The additional 5-min baking was carried out to ensure the stability of defined patterns.
Step 4. Photoresist development: The exposed chip was dipped into MP351 development liquid, diluted with DI water at the ratio of 1:4. The development liquid residue and the photoresist debris were washed out using DI water. Next the cotton swab impregnated with acetone was used to wipe out the photoresist residue at the pattern edges. Finally the developed chip was baked at 120 °C for 5 min.
Step 5. Etching to fabricate electrodes: ITO etching reagent was preheated to 80 °C to shorten etching time. Having been cooled down, the developed chip was dipped into the hot etching reagent for 2 min. The etched chip was washed with DI water to remove remained ITO. All residue photoresist materials were cleaned out by rinsing with acetone, methanol, and then DI water. When the chip was blew with nitrogen and baked to become completely dried, the electrode patterning was finally accomplished.
Step 6. Teflon layer coating: The chip patterned with electrodes was coated with Teflon to form one hydrophobic dielectric layer to finalize the fabrication procedures.
