# Supplementary figures and images for: Development of Coplanar Electro-Wetting Based Microfluidic Sorter to Select Micro-Particles in High Volume Throughput at Milliliter Amount within Twenty Minutes
Source: Sensors (Basel). 2018 Sep 4;18(9):2941. doi: 10.3390/s18092941 (PMC6164432; doi:10.3390/s18092941)

## Slide 1
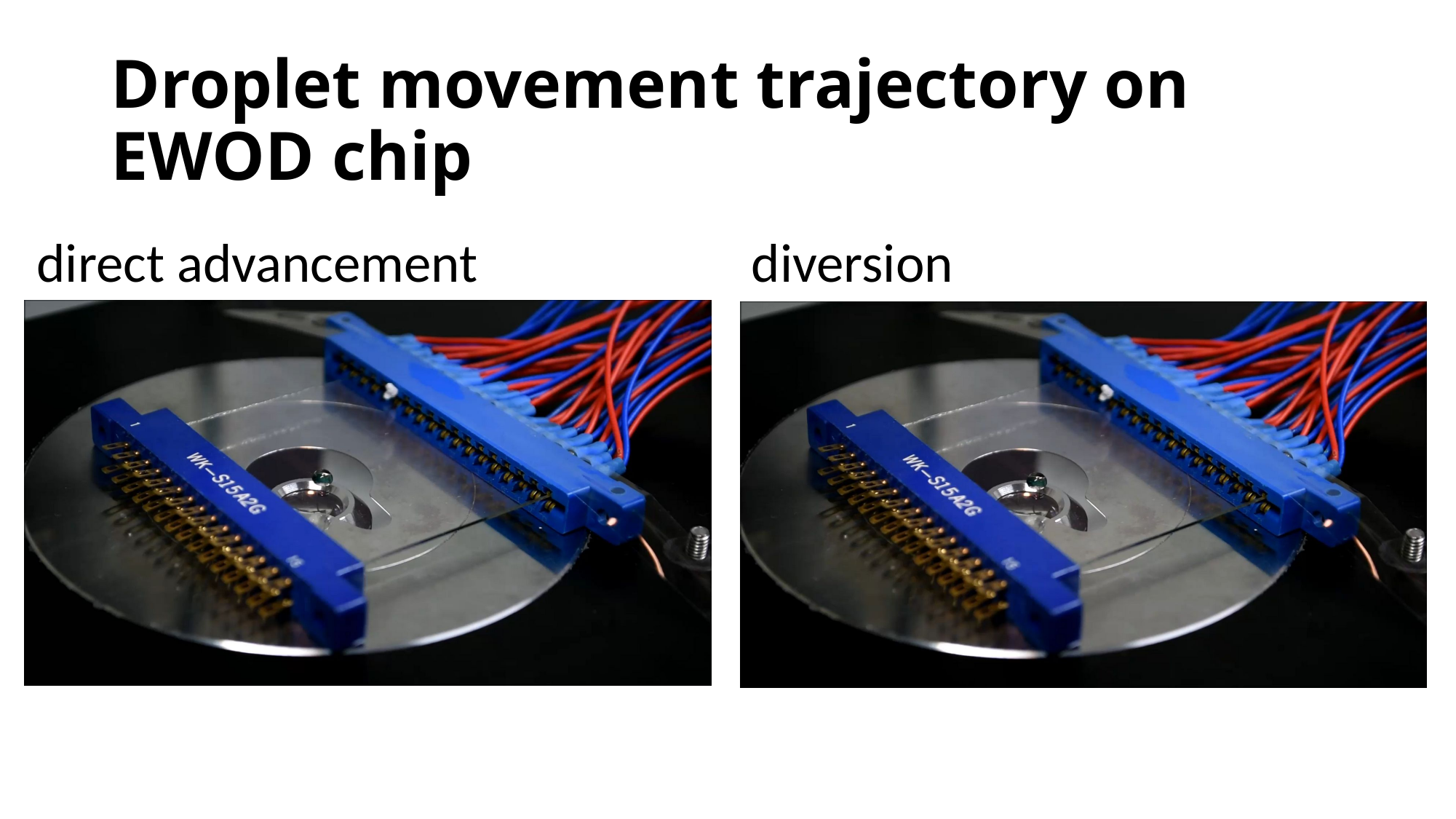

# Droplet movement trajectory on EWOD chip
direct advancement
diversion

Supplement: Supplementary file 1 [file sensors-18-02941-s001.zip › sensors-338985-supplementary.pptx]
